# Bayesian inference for psychology. Part II: Example applications with JASP

**DOI:** 10.3758/s13423-017-1323-7

**Published:** 2017-07-06

**Authors:** Eric-Jan Wagenmakers, Jonathon Love, Maarten Marsman, Tahira Jamil, Alexander Ly, Josine Verhagen, Ravi Selker, Quentin F. Gronau, Damian Dropmann, Bruno Boutin, Frans Meerhoff, Patrick Knight, Akash Raj, Erik-Jan van Kesteren, Johnny van Doorn, Martin Šmíra, Sacha Epskamp, Alexander Etz, Dora Matzke, Tim de Jong, Don van den Bergh, Alexandra Sarafoglou, Helen Steingroever, Koen Derks, Jeffrey N. Rouder, Richard D. Morey

**Affiliations:** 1 Department of Psychological Methods, University of Amsterdam, Nieuwe Achtergracht 129-B, 1018 VZ Amsterdam, The Netherlands; 2 Birla Institute of Technology and Science, Pilani, India; 3 Masaryk University, Brno, Czech Republic; 4 University of California at Irvine, Irvine, CA USA; 5 University of Missouri, Columbia, MO USA; 6 Cardiff University, Cardiff, UK

**Keywords:** Hypothesis test, Statistical evidence, Bayes factor, Posterior distribution

## Abstract

Bayesian hypothesis testing presents an attractive alternative to *p* value hypothesis testing. Part I of this series outlined several advantages of Bayesian hypothesis testing, including the ability to quantify evidence and the ability to monitor and update this evidence as data come in, without the need to know the intention with which the data were collected. Despite these and other practical advantages, Bayesian hypothesis tests are still reported relatively rarely. An important impediment to the widespread adoption of Bayesian tests is arguably the lack of user-friendly software for the run-of-the-mill statistical problems that confront psychologists for the analysis of almost every experiment: the *t*-test, ANOVA, correlation, regression, and contingency tables. In Part II of this series we introduce JASP (http://www.jasp-stats.org), an open-source, cross-platform, user-friendly graphical software package that allows users to carry out Bayesian hypothesis tests for standard statistical problems. JASP is based in part on the Bayesian analyses implemented in Morey and Rouder’s BayesFactor package for R. Armed with JASP, the practical advantages of Bayesian hypothesis testing are only a mouse click away.

As demonstrated in part I of this series, Bayesian inference unlocks a series of advantages that remain unavailable to researchers who continue to rely solely on classical inference (Wagenmakers et al. 2017). For example, Bayesian inference allows researchers to update knowledge, to draw conclusions about the specific case under consideration, to quantify evidence for the null hypothesis, and to monitor evidence until the result is sufficiently compelling or the available resources have been depleted. Generally, Bayesian inference yields intuitive and rational conclusions within a flexible framework of information updating. As a method for drawing scientific conclusions from data, we believe that Bayesian inference is more appropriate than classical inference.

Pragmatic researchers may have a preference that is less pronounced. These researchers may feel it is safest to adopt an inclusive statistical approach, one in which classical and Bayesian results are reported together; if both results point in the same direction this increases one’s confidence that the overall conclusion is robust. Nevertheless, both pragmatic researchers and hardcore Bayesian advocates have to overcome the same hurdle, namely, the difficulty in transitioning from Bayesian theory to Bayesian practice. Unfortunately, for many researchers it is difficult to obtain Bayesian answers to statistical questions for standard scenarios involving correlations, the *t*-test, analysis of variance (ANOVA), and others. Until recently, these tests had not been implemented in any software, let alone user-friendly software. And in the absence of software, few researchers feel enticed to learn about Bayesian inference and few teachers feel enticed to teach it to their students.

To narrow the gap between Bayesian theory and Bayesian practice we developed JASP (JASP Team 2017), an open-source statistical software program with an attractive graphical user interface (GUI). The JASP software package is cross-platform and can be downloaded free of charge from http://www.jasp-stats.org. Originally conceptualized to offer only Bayesian analyses, the current program allows its users to conduct both classical and Bayesian analyses.^1^ Using JASP, researchers can conduct Bayesian inference by dragging and dropping the variables of interest into analysis panels, whereupon the associated output becomes available for inspection. JASP comes with default priors on the parameters that can be changed whenever this is deemed desirable.

This article summarizes the general philosophy behind the JASP program and then presents five concrete examples that illustrate the most popular Bayesian tests implemented in JASP. For each example we discuss the correct interpretation of the Bayesian output. Throughout, we stress the insights and additional possibilities that a Bayesian analysis affords, referring the reader to background literature for statistical details. The article concludes with a brief discussion of future developments for Bayesian analyses with JASP.

## The JASP philosophy

**Fig. 1:**
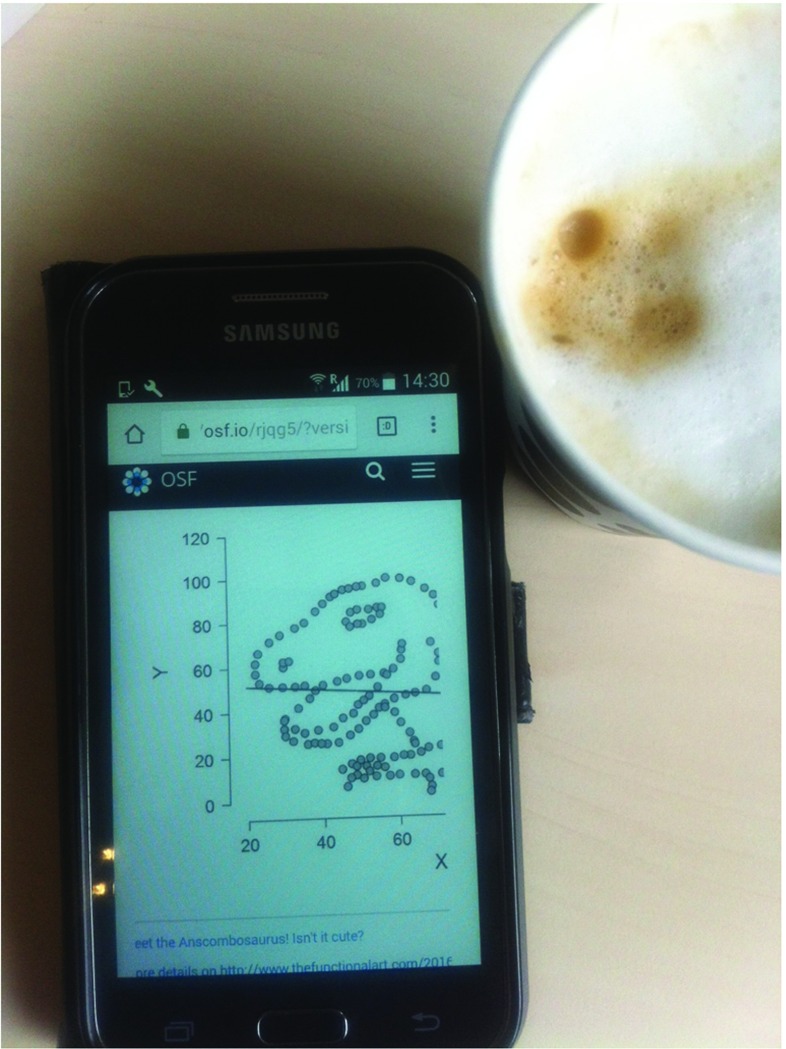
The JASP previewer allows users to inspect the annotated output of a .jasp file on the OSF, even without JASP installed and without an OSF account. The graph shown on the cell phone displays the Anscombosaurus (see http://www.thefunctionalart.com/2016/08/download-datasaurus-never-trust-summary.html). Figure available at https://osf.io/m6bi8/ under under a CC-BY license

The JASP philosophy is based on several interrelated design principles. First, JASP is free and open-source, reflecting our belief that transparency is an essential element of scientific practice. Second, JASP is inferentially inclusive, featuring classical and Bayesian methods for parameter estimation and hypothesis testing. Third, JASP focuses on the statistical methods that researchers and students use most often; to retain simplicity, add-on modules are used to implement more sophisticated and specialized statistical procedures. Fourth, JASP has a graphical user interface that was designed to optimize the user’s experience. For instance, output is dynamically updated as the user selects input options, and tables are in APA format for convenient copy-pasting in text editors such as LibreOffice and Microsoft Word. JASP also uses progressive disclosure, which means that initial output is minimalist and expanded only when the user makes specific requests (e.g., by ticking check boxes). In addition, JASP output retains its state, meaning that the input options are not lost – clicking on the output brings the input options back up, allowing for convenient review, discussion, and adjustment of earlier analyses. Finally, JASP is designed to facilitate open science; from JASP 0.7 onward, users are able to save and distribute data, input options, and output results together as a .jasp file. Moreover, by storing the .jasp file on a public repository such as the Open Science Framework (OSF), reviewers and readers can have easy access to the data and annotated analyses that form the basis of a substantive claim. As illustrated in Fig. 1, the OSF has a JASP previewer that presents the output from a .jasp file regardless of whether the user has JASP installed. In addition, users with an OSF account can upload, download, edit, and sync files stored in their OSF repositories from within JASP. The examples discussed in this article each come with an annotated .jasp file available on the OSF at https://osf.io/m6bi8/. Several analyses are illustrated with videos on the JASP YouTube channel.

The JASP GUI is familiar to users of SPSS and has been programmed in C++, html, and javascript. The inferential engine is based on R (R Development Core Team 2004) and –for the Bayesian analyses– much use is made of the BayesFactor package developed by Morey and Rouder (2015) and the conting package developed by Overstall and King (2014b). The latest version of JASP uses the functionality of more than 110 different R packages; a list is available on the JASP website at https://jasp-stats.org/r-package-list/. The JASP installer does not require that R is installed separately.

Our long-term goals for JASP are two-fold: the primary goal is to make Bayesian benefits more widely available than they are now, and the secondary goal is to reduce the field’s dependence on expensive statistical software programs such as SPSS.

## Example 1: a Bayesian correlation test for the height advantage of US Presidents

For our first example we return to the running example from Part I. This example concerned the height advantage of candidates for the US presidency (Stulp, Buunk, Verhulst, & Pollet, 2013). Specifically, we were concerned with the Pearson correlation *ρ* between the proportion of the popular vote and the height ratio (i.e., height of the president divided by the height of his closest competitor). In other words, we wished to assess the evidence that the data provide for the hypothesis that taller presidential candidates attract more votes. The scatter plot was shown in Figure 1 of Part I. Recall that the sample correlation *r* equaled .39 and was significantly different from zero (*p* = .007, two-sided test, 95% CI [.116,.613]); under a default uniform prior, the Bayes factor equaled 6.33 for a two-sided test and 12.61 for a one-sided test (Wagenmakers et al. 2017).

**Fig. 2:**
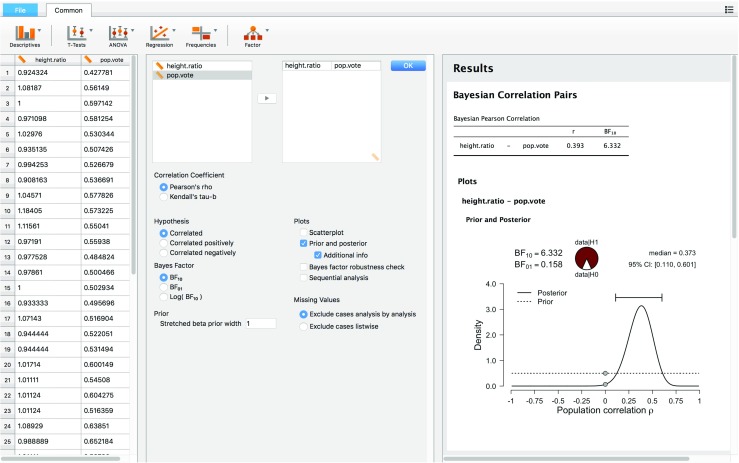
JASP screenshot for the two-sided test for the presence of a correlation between the relative height of the US president and his proportion of the popular vote. The *left panel* shows the data in spreadsheet format; the *middle panel* shows the analysis input options; the *right panel* shows the analysis output

Here we detail how the analysis is conducted in JASP. The left panel of Fig. 2 shows a spreadsheet view of the data that the user has just loaded from a .csv file using the file tab.^2^ Each column header contains a small icon denoting the variable’s measurement level: continuous, ordinal, or nominal (Stevens 1946). For this example, the ruler icon signifies that the measurement level is continuous. When loading a data set, JASP uses a “best guess” to determine the measurement level. The user can click the icon, and change the variable type if this guess is incorrect.

After loading the data, the user can select one of several analyses. Presently the functionality of JASP (version 0.8.1) encompasses the following procedures and tests:

- Descriptives (with the option to display a matrix plot for selected variables).
- Reliability analysis (e.g., Cronbach’s *α*, Gutmann’s *λ*6, and McDonald’s *ω*).
- Independent samples *t*-test, paired samples *t*-test, and one sample *t*-test. Key references for the Bayesian implementation include Jeffreys (1961), Ly, Verhagen, and Wagenmakers (2016a, 2016b), Rouder, Speckman, Sun, Morey, and Iverson (2009) and Wetzels, Raaijmakers, Jakab, and Wagenmakers (2009).
- ANOVA, repeated measures ANOVA, and ANCOVA. Key references for the Bayesian implementation include Rouder, Morey, Speckman, and Province (2012), Rouder, Morey, Verhagen, Swagman, and Wagenmakers (in press), and Rouder, Engelhardt, Mc-Cabe, and Morey (in press).
- Correlation. Key references for the Bayesian implementation include Jeffreys (1961), Ly et al. (2016b), and Ly, Marsman, and Wagenmakers (in press) for Pearson’s *ρ*, and van Doorn, Ly, Marsman, and Wagenmakers (in press) for Kendall’s tau.
- Linear regression. Key references for the Bayesian implementation include Liang, Paulo, Molina, Clyde, and Berger (2008), Rouder and Morey (2012), and Zellner and Siow (1980).
- Binomial test. Key references for the Bayesian implementation include Jeffreys (1961) and O’Hagan and Forster (2004).
- Contingency tables. Key references for the Bayesian implementation include Gunel and Dickey (1974) and Jamil et al. (in press).
- Log-linear regression. Key references for the Bayesian implementation include Overstall and King (2014a) and (2014b).
- Principal component analysis and exploratory factor analysis.

Except for reliability analysis and factor analysis, the above procedures are available both in their classical and Bayesian form. Future JASP releases will expand this core functionality and add logistic regression, multinomial tests, and a series of nonparametric techniques. More specialized statistical procedures will be provided through add-on packages so that the main JASP interface retains its simplicity.

The middle panel of Fig. 2 shows that the user selected a Bayesian Pearson correlation analysis. The two variables to be correlated were selected through dragging and dropping. The middle panel also shows that the user has not specified the sign of the expected correlation under $\mathcal {H}_1$ – hence, JASP will conduct a two-sided test. The right panel of Fig. 2 shows the JASP output; in this case, the user requested and received:

1. The Bayes factor expressed as BF_10_ (and its inverse BF_01_ = 1/BF_10_), grading the intensity of the evidence that the data provide for $\mathcal {H}_1$ versus $\mathcal {H}_0$ (for details see Part I).
2. A proportion wheel that provides a visual representation of the Bayes factor.
3. The posterior median and a 95% credible interval, summarizing what has been learned about the size of the correlation coefficient *ρ* assuming that $\mathcal {H}_1$ holds true.
4. A figure showing (a) the prior distribution for *ρ* under $\mathcal {H}_1$ (i.e., the uniform distribution, which is the default prior proposed by Jeffreys (1961) for this analysis; the user can adjust this default specification if desired), (b) the posterior distribution for *ρ* under $\mathcal {H}_1$, (c) the 95% posterior credible interval for *ρ* under $\mathcal {H}_1$, and (d) a visual representation of the Savage-Dickey density ratio, that is, grey dots that indicate the height of the prior and the posterior distribution at *ρ* = 0 under $\mathcal {H}_1$; the ratio of these heights equals the Bayes factor for $\mathcal {H}_1$ versus $\mathcal {H}_0$ (Dickey & Lientz, 1970; Wagenmakers, Lodewyckx, Kuriyal, & Grasman, 2010).

Thus, in its current state JASP provides a relatively comprehensive overview of Bayesian inference for *ρ*, featuring both estimation and hypothesis testing methods.

Before proceeding we wish to clarify the meaning of the proportion wheel or “pizza plot”. The wheel was added to assist researchers who are unfamiliar with the odds formulation of evidence – the wheel provides a visual impression of the continuous strength of evidence that a given Bayes factor provides. In the presidents example BF_10_ = 6.33, such that the observed data are 6.33 times more likely under $\mathcal {H}_1$ than under $\mathcal {H}_0$. To visualize this ratio, we transform it to the 0-1 interval and plot the resulting magnitude as the proportion of a circle (e.g., Tversky, 1969, Figure 1; Lipkus & Hollands, 1999). For instance, the presidents example has a ratio of BF_10_ = 6.33 and a corresponding proportion of 6.33/7.33 ≈ 0.86;^3^ consequently, the red area (representing the support in favor of $\mathcal {H}_1$) covers 86% of the circle and the white area (representing the support in favor of $\mathcal {H}_0$) covers the remaining 14%.

**Fig. 3:**
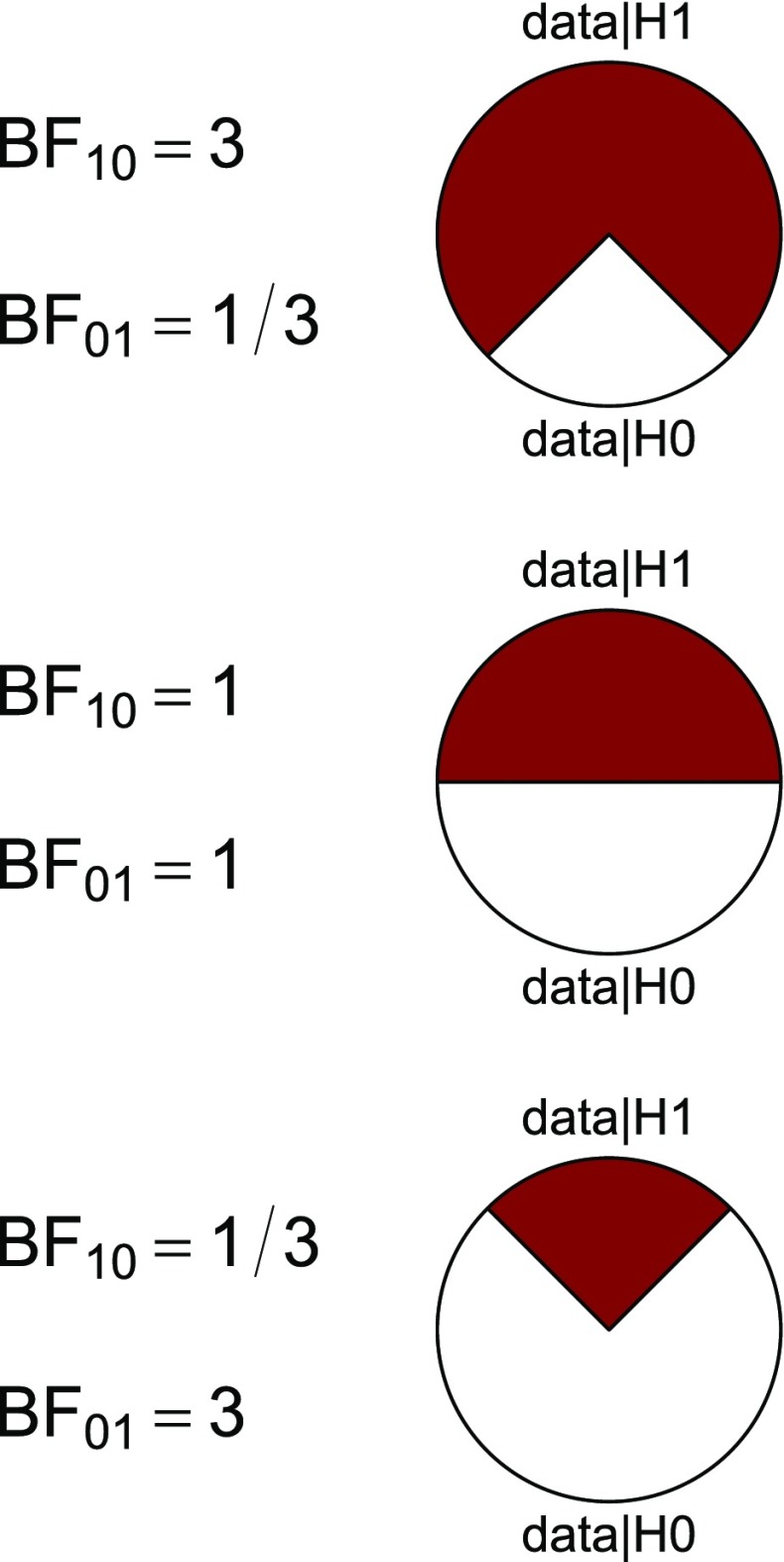
Proportion wheels visualize the strength of evidence that a Bayes factor provides. Ratios are transformed to a magnitude between 0 and 1 and plotted as the proportion of a circular area. Imagine the wheel is a dartboard; you put on a blindfold, the wheel is attached to the wall in random orientation, and you throw darts until you hit the board. You then remove the blindfold and find that the dart has hit the smaller area. How surprised are you? The level of imagined surprise provides an intuition for the strength of a Bayes factor. The analogy is visualized in the Appendix

Figure 3 gives three further examples of proportion wheels. In each panel, the red area represents the support that the data *y* provide for $\mathcal {H}_1$, and the white area represents the complementary support for $\mathcal {H}_0$. Figure 3 shows that when BF_10_ = 3, the null hypothesis still occupies a non-negligible 25% of the circle’s area. The wheel can be used to intuit the strength of evidence even more concretely, as follows. Imagine the wheel is a dart board. You put on a blindfold and the board is attached to a wall in a random orientation. You then throw a series of darts until the first one hits the board. You remove the blindfold and observe that the dart has landed in the smaller area. *How surprised are you?* We propose that this measure of imagined surprise provides a good intuition for degree of evidence that a particular Bayes factor conveys (Jamil, Marsman, Ly, Morey, & Wagenmakers, in press). The top panel of Fig. 3, for instance, represents BF_10_ = 3. Having the imaginary dart land in the white area would be somewhat surprising, but in most scenarios not sufficiently surprising to warrant a strong claim such as the one that usually accompanies a published article. Yet many *p*-values near the .05 boundary (“reject the null hypothesis”) yield evidence that is weaker than BF_10_ = 3 (e.g., Berger & Delampady 1987; Edwards, Lindman, & Savage 1963; Johnson, 2013; Wagenmakers et al., 2017; Wetzels et al., 2011). The dart board analogy is elaborated upon in the Appendix.

The proportion wheel underscores the fact that the Bayes factor provides a graded, continuous measure of evidence. Nevertheless, for historical reasons it may happen that a discrete judgment is desired (i.e., an all-or-none preference for $\mathcal {H}_0$ or $\mathcal {H}_1$). When the competing models are equally likely a priori, then the probability of making an error equals the size of the smaller area. Note that this kind of “error control” differs from that which is sought by classical statistics. In the Bayesian formulation the probability of making an error refers to the individual case, whereas in classical procedures it is obtained as an average across all possible data sets that could have been observed. Note that the long-run average need not reflect the probability of making an error for a particular case (Wagenmakers et al. 2017).

JASP offers several ways in which the present analysis may be refined. In Part I we already showed the results of a one-sided analysis in which the alternative hypothesis $\mathcal {H}_+$ stipulated the correlation to be positive; this one-sided analysis can be obtained by ticking the check box “correlated positively” in the input panel. In addition, the two-sided alternative hypothesis has a default prior distribution which is uniform from − 1 to 1; a user-defined prior distribution can be set through the input field “Stretched beta prior width”. For instance, by setting this input field to 0.5 the user creates a prior distribution with smaller width, that is, a distribution which assigns more mass to values of *ρ* near zero.^4^ Additional check boxes create sequential analyses and robustness checks, topics that will be discussed in the next example.

## Example 2: a Bayesian t-test for a kitchen roll rotation replication experiment

Across a series of four experiments, the data reported in Topolinski and Sparenberg (2012) provided support for the hypothesis that clockwise movements induce psychological states of temporal progression and an orientation toward the future and novelty. Concretely, in their Experiment 2, one group of participants rotated kitchen rolls clockwise, whereas the other group rotated them counterclockwise. While rotating the rolls, participants completed a questionnaire assessing openness to experience. The data from Topolinski and Sparenberg (2012) showed that, in line with their main hypothesis, participants who rotated the kitchen rolls clockwise reported more openness to experience than participants who rotated them counterclockwise (but see Francis, 2013).

**Fig. 4:**
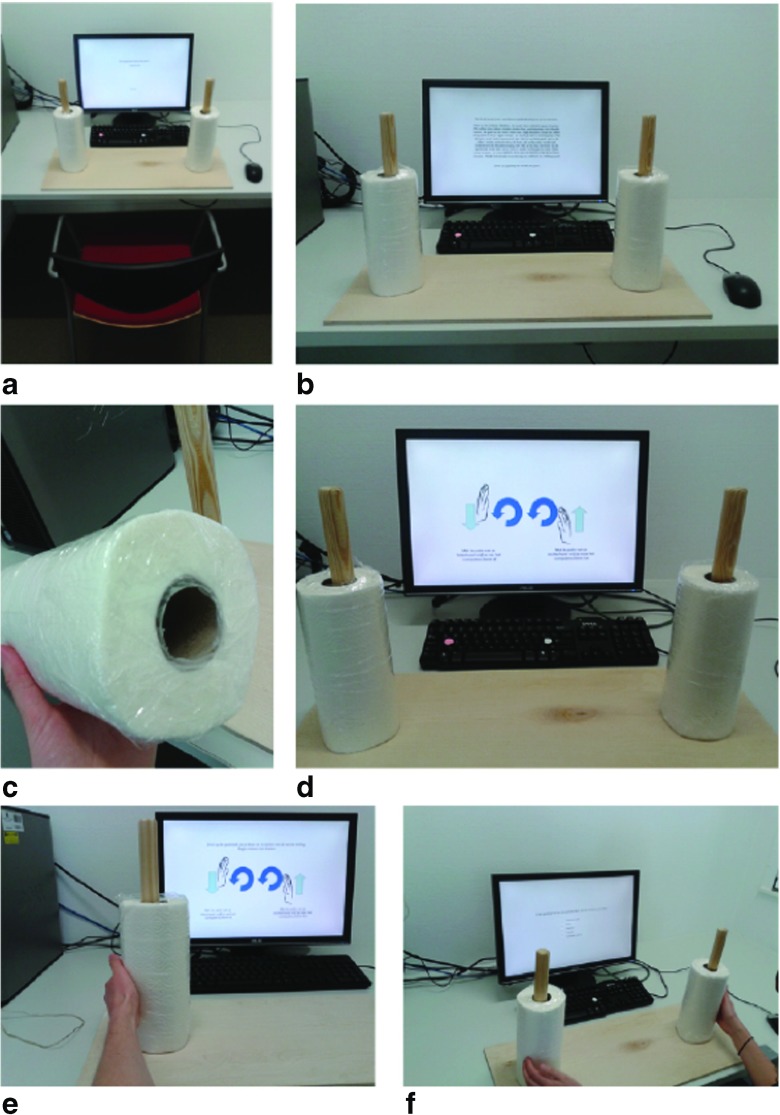
The experimental setting from Wagenmakers et al. (2015): (**a**) the set-up; (**b**) the instructions; (**c**) a close-up of one of the sealed paper towels; (**d**) the schematic instructions; Photos (**e**) and (**f**) give an idea of how a participant performs the experiment. Figure available at https://www.flickr.com/photos/130759277@N05/, under CC license https://creativecommons.org/licenses/by/2.0/

We recently attempted to replicate the kitchen roll experiment from Topolinski and Sparenberg (2012), using a preregistered analysis plan and a series of Bayesian analyses (Wagenmakers et al., 2015, https://osf.io/uszvx/). Thanks to the assistance of the original authors, we were able to closely mimic the setup of the original study. The apparatus and setup for the replication experiment are shown in Fig. 4.

Before turning to aJASP analysis of the data, it is informative to recall the stopping rule procedure specified in the online preregistration form (https://osf.io/p3isc/):

“We will collect aminimum of 20 participants in each between-subject condition (i.e., the clockwise and counterclockwise condition, for aminimum of 40 participants in total). We will then monitor the Bayes factor and stop the experiment whenever the critical hypothesis test (detailed below) reach aBayes factor that can be considered “strong” evidence (Jeffreys 1961); this means that the Bayes factor is either 10 in favor of the null hypothesis, or 10 in favor of the alternative hypothesis. The experiment will also stop whenever we reach the maximum number of participants, which we set to 50 participants per condition (i.e., amaximum of 100 participants in total). Finally, the experiment will also stop on October 1st, 2013. From aBayesian perspective the specification of this sampling plan is needlessly precise; we nevertheless felt the urge to be as complete as possible.”

In addition, the preregistration form indicated that the Bayes factor of interest is the default one-sided *t*-test as specified in Rouder et al. (2009) and Wetzels et al. (2009). The two-sided version of this test was originally proposed by Jeffreys (1961), and it involves a comparison of two hypothesis for effect size *δ*: the null hypothesis $\mathcal {H}_0$ postulates that effect size is absent (i.e., *δ* = 0), whereas the alternative hypothesis $\mathcal {H}_1$ assigns *δ* a Cauchy prior centered on 0 with interquartile range *r* = 1 (i.e., *δ* ∼Cauchy(0,1)). The Cauchy distribution is similar to the normal distribution but has fatter tails; it is a *t*-distribution with a single degree of freedom. Jeffreys chose the Cauchy because it makes the test “information consistent”: with two observations measured without noise (i.e., *y*_1_ = *y*_2_) the Bayes factor in favor of $\mathcal {H}_1$ is infinitely large. The one-sided version of Jeffreys’s test uses a folded Cauchy with positive effect size only, that is, $\mathcal {H}_+: \delta \sim \text {Cauchy}^+(0,1)$.

The specification $\mathcal {H}_+: \delta \sim \text {Cauchy}^+(0,1)$ is open to critique. Some people feel that this distribution is unrealistic because it assigns too much mass to large effect sizes (i.e., 50% of the posterior mass is on values for effect size larger than 1); in contrast, others feel that this distribution is unrealistic because it assigns most mass to values near zero (i.e., *δ* = 0 is the most likely value). It is possible to reduce the value of *r*, and, indeed, the BayesFactor package uses a default value of $r = \frac {1}{2}\sqrt {2} \approx 0.707$, a value that JASP has adopted as well. Nevertheless, the use of a very small value of *r* implies that $\mathcal {H}_1$ and $\mathcal {H}_0$ closely resemble one another in the sense that both models make similar predictions about to-be-observed data; this setting therefore makes it difficult to obtain compelling evidence, especially in favor of a true $\mathcal {H}_0$ (Schönbrodt, Wagenmakers, Zehetleitner, & Perugini, in press). In general, we feel that reducing the value of *r* is recommended if the location of the prior distribution is also shifted away from *δ* = 0. Currently JASP fixes the prior distribution under $\mathcal {H}_1$ to the location *δ* = 0, and consequently we recommend that users deviate from the default setting only when they realize the consequences of their choice.^5^ Note that Gronau, Ly, and Wagenmakers (2017) recently extended the Bayesian *t*-test to include prior distributions on effect size that are centered away from zero. We plan to add these “informed *t*-tests” to JASP in May 2017.

**Fig. 5:**
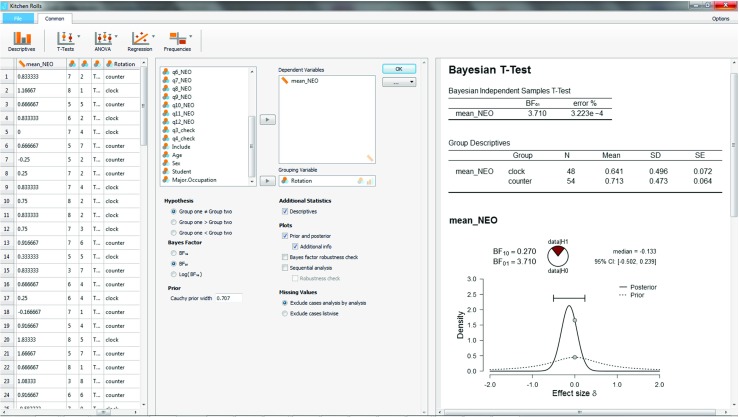
JASP screenshot for the two-sided test of the kitchen roll replication experiment (Wagenmakers et al. 2015). The left panel shows the data in spreadsheet format; the middle panel shows the analysis input options; the right panel shows the analysis output. NB. The “error %” indicates the size of the error in the integration routine relative to the Bayes factor, similar to a coefficient of variation

We are now ready to analyze the data in JASP. Readers who wish to confirm our results can open JASP, go to the File tab, Select “Open”, go to “Examples”, and select the “Kitchen Rolls” data set that is available at https://osf.io/m6bi8/. As shown in the left panel of Fig. 5, the data feature one row for each participant. Each column corresponds to a variable; the dependent variable of interest here is in the column “mean NEO”, which contains the mean scores of each participant on the shortened 12-item version of the openness to experience subscale of the Neuroticism–Extraversion–Openness Personality Inventory (NEO PI-R; Costa & McCrae, 1992; Hoekstra, Ormel, & de Fruyt, 1996). The column “Rotation” includes the crucial information about group membership, with entries either “counter” or “clock”.

In order to conduct the analysis, selecting the “T-test” tab reveals the option “Bayesian Independent Samples T-test”, the dialog of which is displayed in the middle panel of Fig. 5. We have selected “mean NEO” as the dependent variable, and “Rotation” as the grouping variable. After ticking the box “Descriptives”, the output displayed in the right panel of Fig. 5 indicates that the mean openness-to-experience is slightly larger in the counterclockwise group (i.e., *N* = 54;*M* = .71) than in the clockwise group (i.e., *N* = 48; *M* = .64) – note that the effect goes in the direction opposite to that hypothesized by Topolinski and Sparenberg (2012).

For demonstration purposes, at first we refrain from specifying the direction of the test. To contrast our results with those reported by Wagenmakers et al. (2015), we have set the Cauchy prior width to its JASP default *r* = 0.707 instead of Jeffreys’s value *r* = 1. We have also ticked the plotting options “Prior and posterior” and “Additional info”. This produces the plot shown in the right panel of Fig. 5. It is evident that most of the posterior mass is negative. The posterior median is − 0.13, and a 95% credible interval ranges from − 0.50 to 0.23. The Bayes factor is 3.71 in favor of $\mathcal {H}_0$ over the two-sided $\mathcal {H}_1$. This indicates that the observed data are 3.71 times more likely under $\mathcal {H}_0$ than under $\mathcal {H}_1$. Because the Bayes factor favors $\mathcal {H}_0$, in the input panel we have selected “ BF_01_” under “Bayes Factor” – it is easier to interpret BF_01_ = 3.71 than it is to interpret the mathematically equivalent statement BF_10_ = 0.27.

**Fig. 6:**
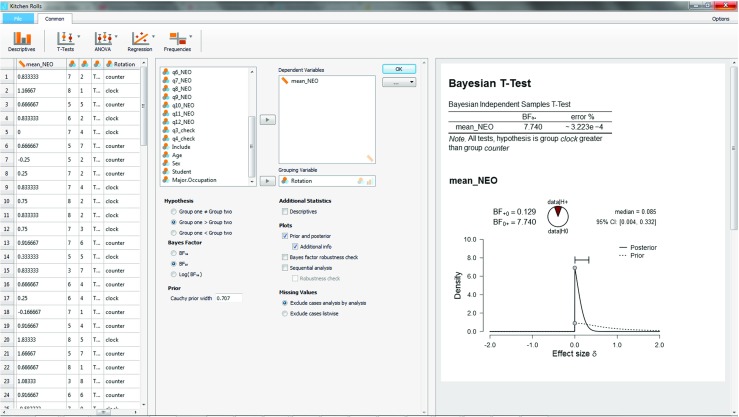
JASP screenshot for the one-sided test of the kitchen roll replication experiment (Wagenmakers et al. 2015). The left panel shows the data in spreadsheet format; the middle panel shows the analysis input options; the right panel shows the analysis output

After this initial investigation we now turn to an analysis of the preregistered order-restricted test (with the exception of using *r* = 0.707 instead of the preregistered *r* = 1). The output of the “Descriptives” option has revealed that “clock” is group 1 (because it is on top), and “counter” is group 2. Hence, we can incorporate the order restriction in our inference by ticking the “Group one > Group two” box under “Hypothesis” in the input panel, as is shown in the middle panel of Fig. 6.

The output for the order-restricted test is shown in the right panel of Fig. 6. As expected, incorporating the knowledge that the observed effect is in the direction opposite to the one that was hypothesized increases the relative evidence in favor of $\mathcal {H}_0$ (see also Matzke et al., 2015). Specifically, the Bayes factor has risen from 3.71 to 7.74, meaning that the observed data are 7.74 times more likely under $\mathcal {H}_0$ than under $\mathcal {H}_+$.

As an aside, note that under $\mathcal {H}_+$ the posterior distribution is concentrated near zero but does not have mass on negative values, in accordance with the order-restriction imposed by $\mathcal {H}_+$. In contrast, the classical one-sided confidence interval ranges from − .23 to *∞*. This classical interval contrasts sharply with its Bayesian counterpart, and, even though the classical interval is mathematically well-defined (i.e., it contains all values that would not be rejected by a one-sided *α* = .05 significance test, see also Wagenmakers et al., 2017), we submit that most researchers will find the classical result neither intuitive nor informative.

**Fig. 7:**
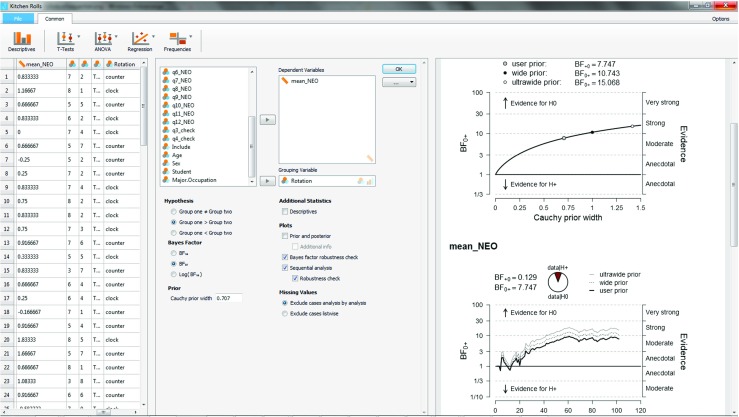
JASP screenshot for the one-sided test of the kitchen roll replication experiment (Wagenmakers et al. 2015). The right panel shows the analysis output: the upper plot is a robustness analysis, and the bottom plot is a sequential analysis combined with a robustness analysis

Next we turn to a robustness analysis and quantify the evidential impact of the width *r* of the Cauchy prior distribution. The middle panel of Fig. 7 shows that the option “Bayes factor robustness check” is ticked, and this produces the upper plot in the right panel of Fig. 7. When the Cauchy prior with *r* equals zero, $\mathcal {H}_1$ is identical to $\mathcal {H}_+$, and the Bayes factor equals 1. As the width *r* increases and $\mathcal {H}_+$ starts to predict that the effect is positive, the evidence in favor of $\mathcal {H}_0$ increases; for the JASP default value *r* = .707, the Bayes factor BF_0+_ = 7.73; for Jeffreys’s default *r* = 1, the Bayes factor BF_0+_ = 10.75; and for the “ultrawide” prior $r = \sqrt {2} \approx 1.41$, the Bayes factor BF_0+_ = 15.04. Thus, over a wide range of plausible values for the prior width *r*, the data provide moderate to strong evidence in favor of the null hypothesis $\mathcal {H}_0$.

Finally, the middle panel of Fig. 7 also shows that the options “Sequential analysis” and “robustness check” are ticked, and these together produce the lower plot in the right panel of Fig. 7. The sequential analysis is of interest here because it was part of the experiment’s sampling plan, and because it underscores how researchers can monitor and visualize the evidential flow as the data accumulate. Closer examination of the plot reveals that for the preregistered value of *r* = 1, Wagenmakers et al. (2015) did not adhere to their preregistered sampling plan to stop data collection as soon as BF_0+_ > 10 or BF_+0_ > 10: after about 55 participants, the dotted line crosses the threshold of BF_0+_ > 10 but data collection nonetheless continued. Wagenmakers et al. (2015, p. 3) explain: “This occurred because data had to be entered into the analysis by hand and this made it more difficult to monitor the Bayes factor continually. In practice, the Bayes factor was checked every few days. Thus, we continued data collection until we reached our predetermined stopping criterion at the point of checking.”

One of the advantages of the sequential robustness plot is that it provides a visual impression of when the Bayes factors for the different priors have converged, in the sense that their difference on the log scale is constant (e.g., Gronau & Wagenmakers, in press). For the current situation, the convergence has occurred after testing approximately 35 participants. To understand why the difference between the log Bayes factors becomes constant after an initial number of observations, consider data *y* that consists of two batches, *y*_1_ and *y*_2_. As mentioned above, from the law of conditional probability we have BF_0+_(*y*) =BF_0+_(*y*_1_) ×BF_0+_(*y*_2_∣*y*_1_). Note that this expression highlights that Bayes factors for different batches of data (e.g., participants, experiments) may not be multiplied blindly; the second factor, BF_0+_(*y*_2_∣*y*_1_), equals the relative evidence from the second batch *y*_2_, after the prior distributions have been properly updated using the information extracted from the first batch *y*_1_ (Jeffreys 1961, p. 333). Rewriting the above expression on the log scale we obtain logBF_0+_(*y*) = logBF_0+_(*y*_1_) + logBF_0+_(*y*_2_∣*y*_1_). Now assume *y*_1_ contains sufficient data such that, regardless of the value of prior width *r* under consideration, approximately the same posterior distribution is obtained. In most situations, this posterior convergence happens relatively quickly. This posterior distribution is then responsible for generating the Bayes factor for the second component, logBF_0+_(*y*_2_∣*y*_1_), and it is therefore robust against differences in *r*.^6^ Thus, models with different values of *r* will make different predictions for data from the first batch *y*_1_. However, after observing a batch *y*_1_ that is sufficiently large, the models have updated their prior distribution to a posterior distribution that is approximately similar; consequently, these models then start to make approximately similar predictions, resulting in a change in the log Bayes factor that is approximately similar as well.

In the first example we noted that the Bayes factor grades the evidence provided by the data on an unambiguous and continuous scale. Nevertheless, the sequential analysis plots in JASP make reference to discrete categories of evidential strength. These categories were inspired by Jeffreys (1961, Appendix B). Table 1 shows the classification scheme used by JASP. We replaced Jeffreys’s labels “worth no more than a bare mention” with “anecdotal” (i.e., weak, inconclusive), “decisive” with “extreme”, and “substantial” with “moderate” (Lee and Wagenmakers 2013); the moderate range may be further subdivided by using “mild” for the 3-6 range and retaining “moderate” for the 6-10 range.^7^ These labels facilitate scientific communication but should be considered only as an approximate descriptive articulation of different standards of evidence. In particular, we may paraphrase Rosnow and Rosenthal (1989) and state that, surely, God loves the Bayes factor of 2.5 nearly as much as he loves the Bayes factor of 3.5.

**Table 1:** A descriptive and approximate classification scheme for the interpretation of Bayes factors BF_10_ (Lee & Wagenmakers 2013; adjusted from Jeffreys 1961)

Bayes factor	Evidence category
> 100	Extreme evidence for $\mathcal {H}_1$
30 - 100	Very strong evidence for $\mathcal {H}_1$
10 - 30	Strong evidence for $\mathcal {H}_1$
3 - 10	Moderate evidence for $\mathcal {H}_1$
1 - 3	Anecdotal evidence for $\mathcal {H}_1$
1	No evidence
1/3 - 1	Anecdotal evidence for $\mathcal {H}_0$
1/10 - 1/3	Moderate evidence for $\mathcal {H}_0$
1/30 - 1/10	Strong evidence for $\mathcal {H}_0$
1/100 - 1/30	Very strong evidence for $\mathcal {H}_0$
< 1/100	Extreme evidence for $\mathcal {H}_0$

## Example 3: a Bayesian one-way ANOVA to test whether pain threshold depends on hair color

**Fig. 8:**
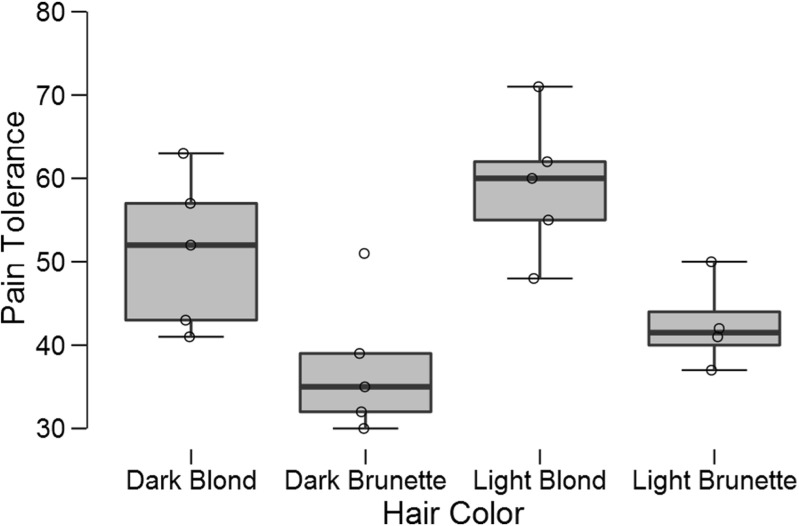
Boxplots and jittered data points for the hair color experiment. Figure created with JASP

An experiment conducted at the University of Melbourne in the 1970s suggested that pain threshold depends on hair color (McClave & Dietrich, 1991, Exercise 10.20). In the experiment, a pain tolerance test was administered to 19 participants who had been divided into four groups according to hair color: light blond, dark blond, light brunette, and dark brunette.^8^ Figure 8 shows the boxplots and the jittered data points. There are visible differences between the conditions, but the sample sizes are small.

**Fig. 9:**
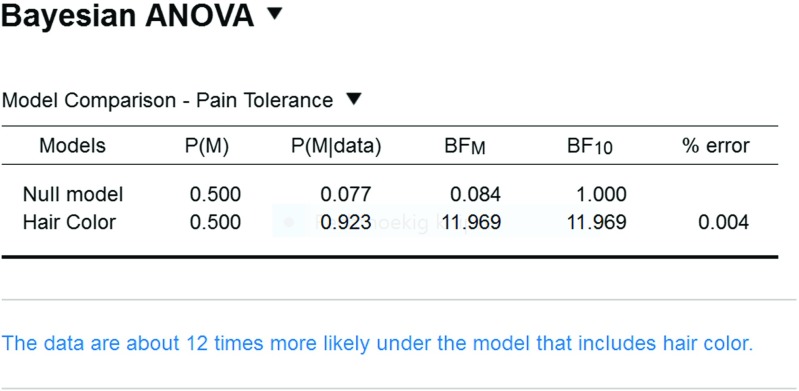
JASP output table for the Bayesian ANOVA of the hair color experiment. The blue text underneath the table shows the annotation functionality that can help communicate the outcome of a statistical analysis

The data may be analyzed with a classical one-way ANOVA. This yields a *p*-value of .004, suggesting that the null hypothesis of no condition differences may be rejected. But how big is the evidence in favor of an effect? To answer this question we now analyze the data in JASP using the Bayesian ANOVA methodology proposed by Rouder et al. (2012) (see also Rouder et al., in press). As was the case for the *t*-test, we assign Cauchy priors to effect sizes. What is new is that the Cauchy prior is now multivariate, and that effect size in the ANOVA model is defined in terms of distance to the grand mean.^9^ The analysis requires that the user opens the data file containing 19 pain tolerance scores in one column and 19 hair colors in the other column. As before, each row corresponds to a participant. The user then selects “ANOVA” from the ribbon, followed by “Bayesian ANOVA”. In the associated analysis menu, the user drags the variable “Pain Tolerance” to the input field labeled “Dependent Variable” and drags the variable “Hair Color” to the input field “Fixed Factors”. The resulting output table with Bayesian results is shown in Fig. 9.

The first column of the output table, “Models”, lists the models under consideration. The one-way ANOVA features only two models: the “Null model” that contains the grand mean, and the “Hair Color” model that adds an effect of hair color. The next point of interest is the “ BF_10_” column; this column shows the Bayes factor for each row-model against the null model. The first entry is always 1 because the null model is compared against itself. The second entry is 11.97, which means that the model with hair color predicts the observed data almost 12 times as well as the null model. As was the case for the output of the *t*-test, the right-most column, “% error”, indicates the size of the error in the integration routine relative to the Bayes factor; similar to a coefficient of variation, this means that small variability is more important when the Bayes factor is ambiguous than when it is extreme.

Column “P(M)” indicates prior model probabilities (which the current version of JASP sets to be equal across all models at hand); column “P(M |data)” indicates the updated probabilities after having observed the data. Column “ BF_M_” indicates the degree to which the data have changed the prior model odds. Here the prior model odds equals 1 (i.e., 0.5/0.5) and the posterior model odds equals almost 12 (i.e., 0.923/0.077). Hence, the Bayes factor equals the posterior odds. JASP offers the user “Advanced Options” that can be used to change the prior width of the Cauchy prior for the model parameters. As the name suggest, we recommend that the user exercises this freedom only in the presence of substantial knowledge of the underlying statistical framework.

Currently JASP does not offer post-hoc tests to examine pairwise differences in one-way ANOVA. Such post-hoc tests have not yet been developed in the Bayesian ANOVA framework. In future work we will examine whether post-hoc tests can be constructed by applying a Bayesian correction for multiple comparisons (i.e., Scott & Berger, 2006, 2010; Stephens & Balding, 2009). Discussion of this topic would take us too far afield.

## Example 4: a Bayesian two-way ANOVA for singers’ height as a function of gender and pitch

**Fig. 10:**
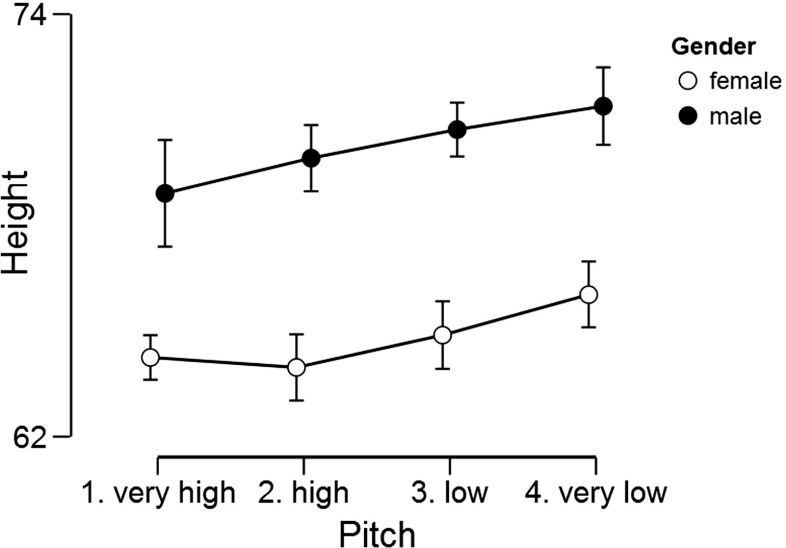
Relation between voice pitch, gender, and height (in inches) for data from 235 singers in the New York Choral Society in 1979. Error bars show 95% confidence intervals. Figure created with JASP

The next data set concerns the heights in inches of the 235 singers in the New York Choral Society in 1979 (Chambers, Cleveland, Kleiner, & Tukey, 1983).^10^ The singers’ voices were classified according to voice part (e.g., soprano, alto, tenor, bass) and recoded to voice pitch (i.e., very low, low, high, very high). Figure 10 shows the relation between pitch and height separately for men and women.

Our analysis concerns the extent to which the dependent variable “height” is associated with gender (i.e., male, female) and/or pitch. This question can be examined statistically using a 2 × 4 ANOVA. Consistent with the visual impression from Fig. 10, a classical analysis yields significant results for both main factors (i.e., *p* < .001 for both gender and pitch) but fails to yield a significant result for the interaction (i.e., *p* = .52). In order to assess the extent to which the data support the presence and absence of these effects we now turn to a Bayesian analysis.

**Fig. 11:**
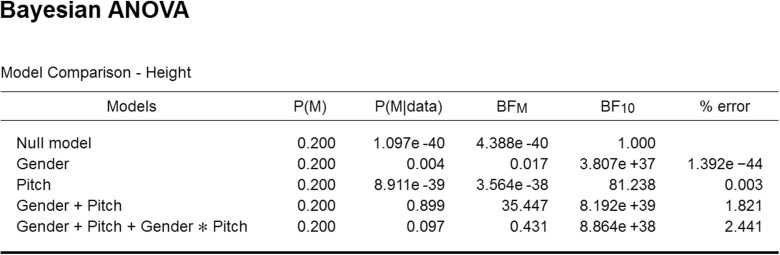
JASP output table for the Bayesian ANOVA of the singers data. Note that JASP uses exponential notation to represent large numbers; for instance, “3.807e +37” represents 3.807 × 10^37^

In order to conduct this analysis in JASP, the user first opens the data set and then navigates to the “Bayesian ANOVA” input panel as was done for the one-way ANOVA. In the associated analysis menu, the user then drags the variable “Height” to the input field labeled “Dependent Variable” and drags the variables “Gender” and “Pitch” to the input field “Fixed Factors”. The resulting output table with Bayesian results is shown in Fig. 11.

The first column of the output table, “Models”, lists the five models under consideration: the “Null model” that contains only the grand mean, the “Gender” model that contains the effect of gender, the “Pitch” model that contains the effect of Pitch, the “Gender + Pitch” model that contains both main effects, and finally the “Gender + Pitch + Gender × Pitch” model that includes both main effects and the interaction. Consistent with the principle of marginality, JASP does not include interactions in the absence of the component main effects; for instance, the interaction-only model “Gender × Pitch” may not be entertained without also adding the two main effects (for details, examples, and rationale see Bernhardt & Jung, 1979, Griepentrog, Ryan, & Smith 1982, McCullagh & Nelder, 1989; Nelder, 1998, 2000; Peixoto, 1987, 1990; Rouder, Engelhardt, et al., in press; Rouder, Morey, et al., in press; Venables, 2000).

Now consider the BF _10_ column. All models (except perhaps for Pitch) receive overwhelming evidence in comparison to the Null model. The model that outperforms the Null model the most is the two main effects model, Gender + Pitch. Adding the interaction makes the model less competitive. The evidence against including the interaction is roughly a factor of ten. This can be obtained as 8.192e+39 / 8.864e+38 ≈ 9.24. Thus, the data are 9.24 times more likely under the two main effects model than under the model that adds the interaction.

Column “P(M)” indicates the equal assignment of prior model probability across the five models; column “P(M |data)” indicates the posterior model probabilities. Almost all posterior mass is centered on the two main effects model and the model that also includes the interaction. Column “BF _M_” indicates the change from prior to posterior model odds. Only the two main effects model has received support from the data in the sense that the data have increased its model probability.

**Fig. 12:**
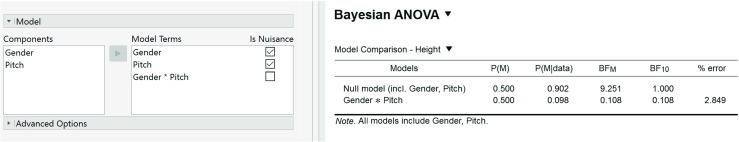
JASP screenshot and output table for the Bayesian ANOVA of the singers data, with Gender and Pitch added as nuisance factors

Above we wished to obtain the Bayes factor for the main effects only model versus the model that adds the interaction. We accomplished this objective by comparing the strength of the Bayes factor against the Null model for models that exclude or include the critical interaction term. However, this Bayes factor can also be obtained directly. As shown in Fig. 12, the JASP interface allows the user to specify Gender and Pitch as nuisance variables, which means that they are included in every model, including the Null model. The Bayes factor of interest is BF_10_ = 0.108; when inverted, this yields BF _01_ = 1/0.108 = 9.26, confirming the result obtained above through a simple calculation. The fact that the numbers are not identical is due to the numerical approximation; the error percentage is indicated in the right-most column.

In sum, the Bayesian ANOVA reveals that the data provide strong support for the two main effects model over any of the simpler models. The data also provide good support against including the interaction term.

Finally, as described in Cramer et al. (2016), the multiway ANOVA harbors a multiple comparison problem. As for the one-way ANOVA, this problem can be addressed by applying the proper Bayesian correction method (i.e., Scott & Berger 2006, 2010; Stephens & Balding,^2009^). This correction has not yet been implemented in JASP.

## Example 5: a Bayesian two-way repeated measures ANOVA for people’s hostility towards arthropods

**Fig. 13:**
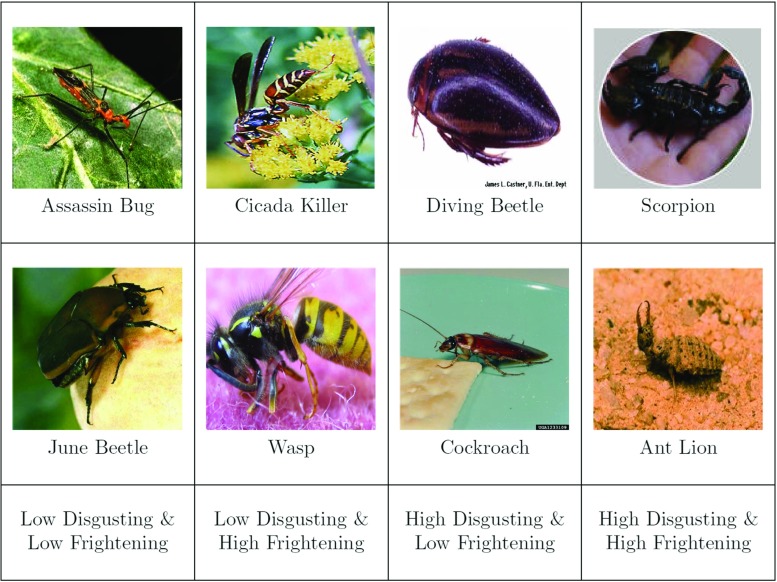
The arthropod stimuli used in Ryan and Wilde (2013). Each cell in the 2 × 2 repeated measures design contains two arthropods. The original stimuli did not show the arthropod names. Figure adjusted from Ryan and Wilde (2013)

In an online experiment, Ryan, Wilde, and Crist (2013) presented over 1300 participants with pictures of eight arthropods. For each arthropod, participants were asked to rate their hostility towards that arthropod, that is, “...the extent to which they either wanted to kill, or at least in some way get rid of, that particular insect” (p. 1297). The arthropods were selected to vary along two dimensions with two levels: disgustingness (i.e., low disgusting and high disgusting) and frighteningness (i.e., low frighteningness and high frighteningness). Figure 13 shows the arthropods and the associated experimental conditions. For educational purposes, we ignore the gender factor, we ignore the fact that the ratings are not at all normally distributed, we analyze data from a subset of 93 participants, and we side-step the nontrivial question of whether to model the item-effects. The pertinent model is a linear mixed model, and the only difference with respect to the previous example is that we now require a prior for the new random factor –in this case, participants– which is set a little wider because we assume a priori that participants are variable in the main effect (for an in-depth discussion see Rouder et al., in press).

**Fig. 14:**
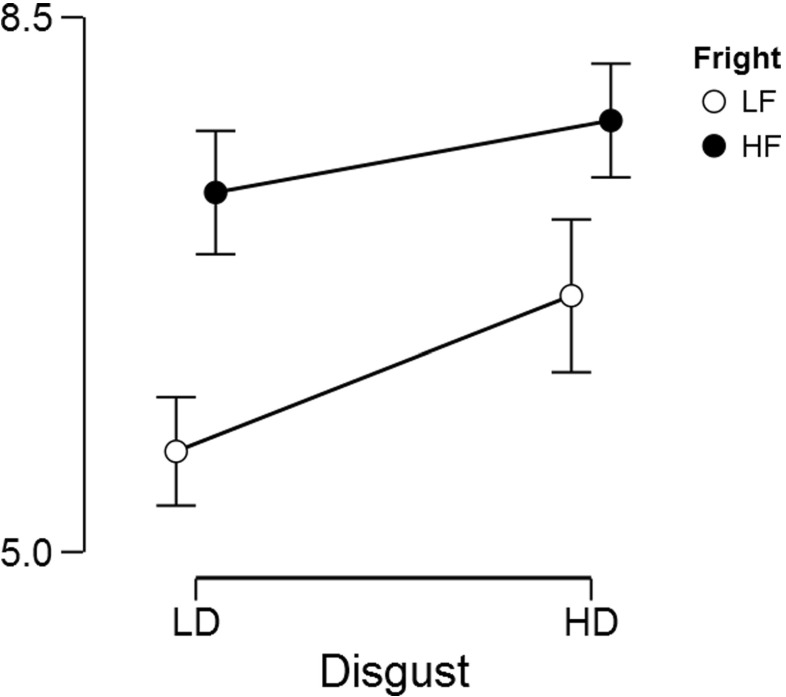
Hostility ratings for arthropods that differ in disgustingness (i.e., LD for low disgusting and HD for high disgusting) and frighteningness (i.e., LF for low frighteningness and HF for high frighteningness). Error bars show 95% confidence intervals. Data kindly provided by Ryan and Wilde (2013). Figure created with JASP

Our analysis asks whether and how people’s hostility towards arthropods depends on their disgustingness and frighteningness. As each participant’s rated all eight arthropods, these data can be analyzed using a repeated measures 2 × 2 ANOVA. A classical analysis reveals that the main effects of disgustingness and frighteningness are both highly significant (i.e., *p*’s < .001) whereas the interaction is not significant (*p* = 0.146). This is consistent with the data as summarized in Fig. 14: arthropods appear to be particularly unpopular when they are high rather than low in disgustingness, and when they are high rather than low in frighteningness. The data do not show a compelling interaction. To assess the evidence for and against the presence of these effects we now turn to a Bayesian analysis.

To conduct the Bayesian analysis the user first needs to open the data set in JASP.^11^ Next the user selects the “Bayesian Repeated Measures ANOVA” input panel that is nested under the ribbon option “ANOVA”. Next the user needs to name the factors (here “Disgust” and “Fright”) and their levels (here “LD”, “HD”, and “LF”, “HF”). Finally the input variables need to be dragged to the matching “Repeated Measures Cells”.

**Fig. 15:**
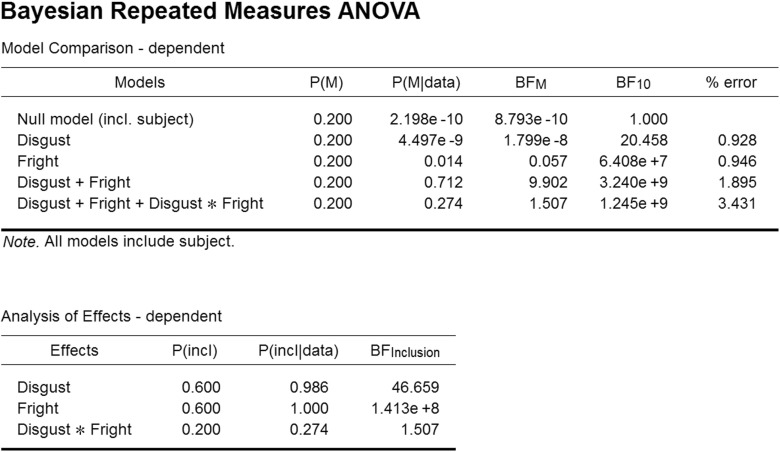
JASP screenshot for the output tables of the Bayesian ANOVA for the arthropod experiment. The top table shows the model-based analysis, whereas the bottom panels shows the analysis of effects, averaging across the models that contain a specific factor. See text for details

The analysis produces the output shown in the top panel of Fig. 15. As before, the column “Models” lists the five different models under consideration. The BF_10_ column shows that compared to the Null model, all other models (except perhaps the Disgust-only model) receive overwhelming support from the data. The model that receives the most support against the Null model is the two main effects model, Disgust + Fright. Adding the interaction decreases the degree of this support by a factor of 3.240/1.245 = 2.6. This is the Bayes factor in favor of the two main effects model versus the model that also includes the interaction. The same result could have been obtained directly by adding “Disgust” and “Fright” as nuisance variables, as was illustrated in the previous example.

The “P(M)” column shows the uniform distribution of prior model probabilities across the five candidate models, and the “P(M |data)” column shows the posterior model probabilities. Finally, the “BF _M_” column shows the change from prior model odds to posterior model odds. This Bayes factor also favors the two main effects model, but at the same time indicates mild support in favor of the interaction model. The reason for this discrepancy (i.e., a Bayes factor of 2.6 against the interaction model versus a Bayes factor of 1.5 in favor of the interaction model) is that these Bayes factors address different questions: The Bayes factor of 2.6 compares the interaction model against the two main effects model (which happens to be the model that is most supported by the data), whereas the Bayes factor of 1.5 compares the interaction model against all candidate models, some of which receive almost no support from the data. Both analyses are potentially of interest. Specifically, when the two main effects model decisively outperforms the simpler candidate models then it may be appropriate to assess the importance of the interaction term by comparing the two main effects model against the model that adds the interaction. However, it may happen that the simpler candidate models outperform the two main effects model – in other words, the two main effects model has predicted the data relatively poorly compared to the Null model or one of the single main effects models. In such situations it is misleading to test the importance of the interaction term by solely focusing on a comparion to the poorly performing two main effects model. In general we recommend radical transparency in statistical analysis; an informative report may present the entire table shown in Fig. 15. In this particular case, both Bayes factors (i.e., 2.6 against the interaction model, and 1.5 in favor of the interaction model) are “not worth more than a bare mention” (Jeffreys 1961, Appendix B); moreover, God loves these Bayes factors almost an equal amount, so it may well be argued that the discrepancy here is more apparent than real.

As the number of factors grows, so does the number of models. With many candidate models in play, it may be risky to base conclusions on a comparison involving a small subset. In Bayesian model averaging (BMA; e.g., Etz & Wagenmakers, in press; Haldane 1932; Hoeting, Madigan, Raftery, & Volinsky, 1999) the goal is to retain model selection uncertainty by averaging the conclusions from each candidate model, weighted by that model’s posterior plausibility. In JASP this is accomplished by ticking the “Effects” input box, which results in an output table shown in the bottom panel of Fig. 15.

In our example, the averaging in BMA occurs over the models shown in the Model Comparison table (top panel of Fig. 15). For instance, the factor “Disgust” features in three models (i.e., Disgust only, Disgust + Fright, and Disgust + Fright + Disgust * Fright). Each model has a prior model probability of 0.2, so the summed prior probability of the three models that include disgust equals 0.6; this is known as the prior inclusion probability for Disgust (i.e., the column P(incl)). After the data are observed we can similarly consider the sum of the posterior model probabilities for the models that include disgust, yielding 4.497e-9 + 0.712 + 0.274 = 0.986. This is the posterior inclusion probability (i.e., column P(incl |data)). The change from prior to posterior inclusion odds is given in the column “BF _Inclusion_”. Averaged across all candidate models, the data strongly support inclusion of both main factors Disgust and Fright. The interaction only receives weak support. In fact, the interaction term occurs only in a single model, and therefore its posterior inclusion probability equals the posterior model probability of that model (i.e., the one that contains the two main effects and the interaction).

It should be acknowledged that the analysis of repeated measures ANOVA comes with a number of challenges and caveats. The development of Bayes factors for crossed-random effect structures is still a topic of ongoing research. And in general, JASP currently does not feature an extensive suite of estimation routines to assess the extent to which generic model assumptions (e.g., sphericity) are violated.

## Future directions for Bayesian analyses in JASP

The present examples provides a selective overview of default Bayesian inference in the case of the correlation test, *t*-test, one-way ANOVA, two-way ANOVA, and two-way repeated measures ANOVA. In JASP, other analyses can be executed in similar fashion (e.g., for contingency tables, Jamil, Ly, et al., in press, Jamil, Marsman, et al., in press; Scheibehenne, Jamil, & Wagenmakers, in press; or for linear regression Rouder & Morey, 2012). A detailed discussion of the entire functionality of JASP is beyond the scope of this article.

In the near future, we aim to expand the Bayesian repertoire of JASP, both in terms of depth and breadth. In terms of depth, our goal is to provide more and better graphing options, more assumption tests, more nonparametric tests, post-hoc tests, and corrections for multiplicity. In terms of breadth, our goal is to include modules that offer the functionality of the BAS package (i.e., Bayesian model averaging in regression, Clyde, 2016), the informative model comparison approach (e.g., Gu, Mulder, Decović, & Hoijtink, 2014; Gu, 2016; Mulder, 2014, 2016), and a more flexible and subjective prior specification approach (e.g., Dienes, 2011, 2014, 2016; Gronau et al., 2017). By making the additional functionality available as add-on modules, beginning users are shielded from the added complexity that such options add to the interface. In the short-term we also aim to develop educational materials that make JASP output easier to interpret and to teach to undergraduate students. This entails writing a JASP manual, developing course materials, writing course books, and designing a Massive Open Online Course.

Our long-term goal is for JASP to facilitate several aspects of statistical practice. Free and user-friendly, JASP has the potential to benefit both education and research. By featuring both classical and Bayesian analyses, JASP implicitly advocates a more inclusive statistical approach. JASP also aims to assist with data preparation and aggregation; currently, this requires that JASP launches and interacts with an external editor (see our data-editing video at https://www.youtube.com/watch?v=1dT-iAU9Zuc&t=70s); in the future, JASP will have its own editing functionality including filtering and outlier exclusion. Finally, by offering the ability to save, annotate, and share statistical output, JASP promotes a transparent way of communicating one’s statistical results. An increase in statistical transparency and inclusiveness will result in science that is more reliable and more replicable.

As far as the continued development of JASP is concerned, our two main software developers and several core team members of the JASP team have tenured positions. The Psychological Methods Group at the University of Amsterdam is dedicated to long-term support for JASP, and in 2017 we have received four million euro to set up projects that include the development of JASP as a key component. The JASP code is open-source and will always remain freely available online. In sum, JASP is here to stay.

## Concluding comments

In order to promote the adoption of Bayesian procedures in psychology, we have developed JASP, a free and open-source statistical software program with an interface familiar to users of SPSS. Using JASP, researchers can obtain results from Bayesian techniques easily and without tears. Dennis Lindley once said that “Inside every Non-Bayesian, there is a Bayesian struggling to get out” (Jaynes 2003). We hope that software programs such as JASP will act to strengthen the resolve of one’s inner Bayesian and pave the road for a psychological science in which innovative hypotheses are tested using coherent statistics.

## Figures and Tables

**Fig. 16:**
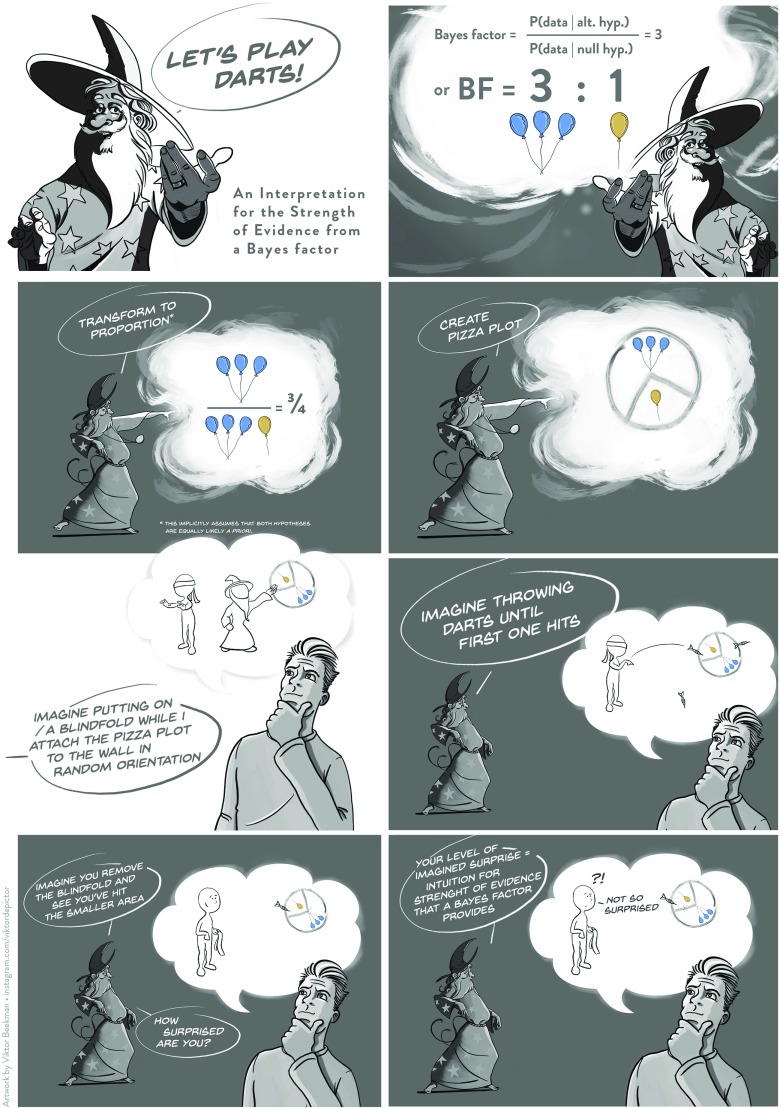
A dart board analogy to intuit the strength of evidence that a Bayes factor provides. Figure available at https://osf.io/m6bi8/ under under a CC-BY license

## Footnotes

1 Bayesian advocates may consider the classical analyses a Bayesian Trojan horse.

2 JASP currently reads the following file formats: .jasp, .txt, .csv (i.e., a plain text file with fields separated by commas), .ods (i.e., OpenDocument Spreadsheet, a file format used by OpenOffice), and .sav (i.e., the SPSS file format).

3 With equal prior odds, a ratio of *x* corresponds to a proportion of *x*/(*x* + 1).

4 Statistical detail: the stretched beta prior is a beta(*a*,*a*) distribution transformed to cover the interval from − 1 to 1. The prior width is defined as 1/*a*. For instance, setting the stretched beta prior width equal to 0.5 is conceptually the same as using a beta(2, 2) distribution on the 0-1 interval and then transforming it to cover the interval from − 1 to 1, such that it is then symmetric around *ρ* = 0.

5 For an indication of how Bayes factors can be computed under any proper prior distribution see http://jeffrouder.blogspot.nl/2016/01/what-priors-should-i-use-part-i.html, also available as a pdf file at the OSF project page https://osf.io/m6bi8/.

6 This also suggests that one can develop a Bayes factor that is robust against plausible changes in *r*: first, sacrifice data *y*_1_ until the posterior distributions are similar; second, monitor and report the Bayes factor for the remaining data *y*_2_. This is reminiscent of the idea that underlies the so-called intrinsic Bayes factor (Berger and Pericchi 1996), a method that also employs a “training sample” to update the prior distributions before the test is conducted using the remaining data points. The difference is that the intrinsic Bayes factor selects a training sample of minimum size, being just large enough to identify the model parameters.

7 The present authors are not all agreed on the usefulness of such descriptive classifications of Bayes factors. All authors agree, however, that the advantage of Bayes factors is that –unlike for instance *p* values which are dichotomized into “significant” and “non-significant”– the numerical value of the Bayes factor can be interpreted directly. The strength of the evidence is not dependent on any conventional verbal description, such as “strong”.

8 The data are available at http://www.statsci.org/data/oz/blonds.html.

9 The Cauchy prior width *r*_*t*_ for the independent samples *t*-tests yields the same result as a two-group one-way ANOVA with a fixed effect scale factor *r*_*A*_ equal to $r_t/\sqrt {2}$. With the default setting $r_t=1/2 \cdot \sqrt {2}$, this produces *r*_*A*_ = 0.5. In sum, for the default prior settings in JASP the independent samples *t*-test and the two-group one-way ANOVA yield the same result. For examples see https://cran.r-project.org/web/packages/BayesFactor/vignettes/priors.html.

10 Data available at https://stat.ethz.ch/R-manual/R-devel/library/lattice/html/singer.html https://stat.ethz.ch/R-manual/R-devel/library/lattice/html/singer.html.

11 The data set is available on the project OSF page and from within JASP (i.e., File → Open → Examples → Bugs).
